# Leishmania life cycle images in the cutaneous cytologic smear of an immunocompetent patient

**DOI:** 10.4103/0970-9371.66695

**Published:** 2010-01

**Authors:** Roberta Zappacosta, Roberta Claudi, Salvatore Magnasco, Emma Dell’osa

**Affiliations:** Surgical Pathology Unit, Oncology and Neurosciences Department, Section of Cytopathology “G. d’Annunzio”, University of Chieti-Pescara, Via Dei Vestini, Chieti, Italy

**Keywords:** Cutaneous leishmaniasis, leishmania, sporadic leishmaniasis, scrape cytology

## Abstract

Cutaneous leishmania life cycle images on cytology smears are very rare. We report herein a gallery of cytologic images from a case of sporadic cutaneous leishmaniasis in a 61 year old man presenting with ulcerative skin lesion.

## Introduction

Leishmaniasis represents a group of infections caused by flagellate protozoans of the genus *Leishmania*. Leishmania species are transmitted to humans by the bite of infected female Phlebotomus sand-flies. Leishmaniasis can be divided into cutaneous and visceral clinical categories.[1,2] Cutaneous leishmaniasis is characterized by localized skin lesions, which can become chronic with disfiguring scars. Despite its increasing worldwide incidence, cutaneous leishmaniasis is considered to be one of the so-called neglected diseases because of its favorable and rarely fatal outcome.[3] Methods for parasitological diagnosis include *in vitro* culture of infected tissue or inoculation into animals. Species identification can be accomplished by isoenzyme analysis of cultured promastigotes, by molecular methods or by monoclonal antibody investigation. Routine blood tests have no role in the diagnosis of leishmaniasis. Serological testing is not always helpful because the antibodies are undetectable or are at low levels.[4] Moreover, modern immunodiagnostic methods are costly and their availability is limited in clinical routine.

Then, although many sophisticated studies have been performed in order to clarify Leishmania’s replication and transmission model, ideally, all cases of leishmaniasis should be confirmed by morphological demonstration of the parasite in the host. Histological examination still represents the most commonly used technique to visualize the parasite. Cutaneous leishmania life cycle images in cytology slides stained with papanicolaou procedure are very rare. The main purpose of this report is to make such images available.

## Case Report

A 61-year-old immunocompetent Italian man, born and residing in Abruzzo, presented to our observation because of the persistence of multiple ulcerative nodules on the skin of his right cheek (larger nodule measured 2 cm in maximum diameter), partially covered by scaly scabs.

**Figure 1:**
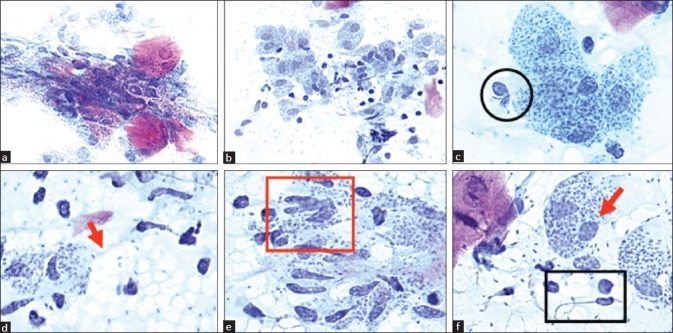
Cytology smear showing cutaneous leishmaniasis (Papanicolaou stain). (a, b) Promastigotes migrating towards cutaneous cells (×400 and ×200 respectively). (c) Promastigotes actively invading neutrophil granulocytes (black circle) (×630). (d) Intact macrophages filled with amastigotes having clearly visible nuclei and rhizoplast (red arrow) (×1,000). (e) Amastigotes leaving infected cells (red square). (f) Amastigotes showing kinetoplast (red arrow) (×1,000). Promastigotes, one of which has an abnormally long flagella (black rectangle), indicative of failed cell division (×1,000)

A complete blood picture was normal. The patient referred no travels and had received several topical treatments (antibiotics, antimycotics, steroids), which had failed. After cleaning off the excess blood, cytology smears were performed by sampling cells from the ulcer with a sterile lancet and by smearing the sample directly onto glass slides. Pathological examination showed the presence of a large number of intra- and extracellular Leishmania promastigotes and a massive histiocyte infiltration, laden with amastigotes (Leishman-Donovan bodies) [Figure 1].

## Discussion

Amastigotes represent the host form of Leishmania. To ensure that the visualized structures are amastigotes rather than other organisms (Histoplasma spp), one should look for the characteristic size (2–4 *μ*m in diameter), shape (round or oval) and internal organelles (the nucleus and the kinetoplast). Particularly, the kinetoplast (a specialized mitochondrial structure containing extranuclear DNA) should be visualized. Other disorders mimicking cutaneous leishmaniasis include traumatic ulcerative lesions, foreign body reactions, infected insect bites, impetigo, fungal and mycobacterial infections, sarcoidosis and neoplasms. Delays in diagnosis can lead to diagnostic difficulties (due to the paucity of the parasite in the host), to bigger lesions and scars and to the occurrence of bacterial infection and mucosal individualized.

Leishmaniasis is a curable disease but antileishmaniasis therapy is frequently subjective. The overabundance of published reports repeatedly creates the false impression that many good treatment options exist. The reality is that few drugs have been assessed adequately in clinical trials and that the management of cutaneous leishmaniasis is often complicated by rapid self-healing. Certainly, the management of each patient’s case should be individualized.[6]

## Conclusions

It is our opinion that cytological examination is painless, useful and effective diagnostic tool in the diagnosis of cutaneous leishmaniasis and would lead to initiation of appropriate therapy.
